# 1,3-Bis(6-methyl­pyridin-2-yl)-1*H*-imidazol-3-ium hexa­fluoro­phosphate

**DOI:** 10.1107/S1600536813006971

**Published:** 2013-03-16

**Authors:** Hoyeon Bae, Minyoung Yoon, Ho-Jung Sun, Dong-Heon Lee, Gyungse Park

**Affiliations:** a Department of Chemistry, Chonbuk National University, Jeonju, Chonbuk 561-756, Republic of Korea; b Center for Smart Supramolecules, Department of Chemistry and Division of Advanced Materials Science, Pohang University of Science and Technology, Pohang 790-784, Republic of Korea; c Department of Material Science & Engineering, Kunsan National University, Jeonbuk 573-701, Republic of Korea; d Department of Chemistry and Research Institute of Physics and Chemistry, Chonbuk National University, Jeonju 561-756, Republic of Korea; e Department of Chemistry, Kunsan National University, Kunsan, Chonbuk 573-701, Republic of Korea

## Abstract

In the title salt, C_17_H_19_N_4_^+^·PF_6_^−^, the two pyridine rings of the cation are inclined to one another by 15.89 (8)°, and inclined to the imidazole ring by 65.05 (10) and 64.07 (10)°. In the crystal, the cations and anions are linked *via* a series of C—H⋯N and C—H⋯F hydrogen bonds, forming two-dimensional networks lying parallel to (001).

## Related literature

For the isolation of an *N*-heterocyclic carbene, see: Arduengo *et al.* (1991 ▶). For related structures, see: Huang *et al.* (2011 ▶); Grieco *et al.* (2011 ▶); Kim *et al.* (2009 ▶). For applications of *N*-heterocyclic carbenes in catalytic processes, see: Enders *et al.* (1996 ▶); Frenzel *et al.* (1999 ▶); Scholl *et al.* (1999 ▶).
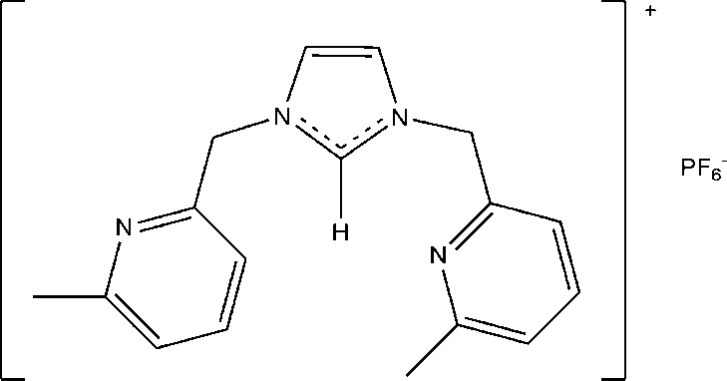


## Experimental

### 

#### Crystal data

C_17_H_19_N_4_^+^·PF_6_^−^
*M* *_r_* = 424.33
Triclinic, 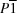

*a* = 6.3839 (3) Å
*b* = 12.0353 (5) Å
*c* = 12.8006 (5) Å
α = 108.039 (2)°
β = 96.091 (2)°
γ = 100.593 (2)°
*V* = 905.12 (7) Å^3^
*Z* = 2
Mo *K*α radiation
μ = 0.22 mm^−1^
*T* = 100 K
0.16 × 0.07 × 0.07 mm

#### Data collection

Bruker APEXII diffractometer
Absorption correction: multi-scan (*SADABS*; Sheldrick, 1996 ▶) *T*_min_ = 0.965, *T*_max_ = 0.986
19206 measured reflections
3700 independent reflections
2898 reflections with *I* > 2σ(*I*)
*R*_int_ = 0.032

#### Refinement

*R*[*F* ^2^ > 2σ(*F* ^2^)] = 0.039
*wR*(*F* ^2^) = 0.133
*S* = 0.97
3700 reflections
269 parameters
H-atom parameters constrained
Δρ_max_ = 0.35 e Å^−3^
Δρ_min_ = −0.45 e Å^−3^

### 

Data collection: *APEX2* (Bruker, 2000 ▶); cell refinement: *SAINT* (Bruker, 2000 ▶); data reduction: *SAINT*; program(s) used to solve structure: *SHELXS97* (Sheldrick, 2008 ▶); program(s) used to refine structure: *SHELXL97* (Sheldrick, 2008 ▶); molecular graphics: *DIAMOND* (Brandenburg, 1999 ▶); software used to prepare material for publication: *SHELXL97*.

## Supplementary Material

Crystal structure: contains datablock(s) I, global. DOI: 10.1107/S1600536813006971/ng5317sup1.cif

Structure factors: contains datablock(s) I. DOI: 10.1107/S1600536813006971/ng5317Isup2.hkl

Supplementary material file. DOI: 10.1107/S1600536813006971/ng5317Isup3.cml

Additional supplementary materials:  crystallographic information; 3D view; checkCIF report

## Figures and Tables

**Table 1:** Hydrogen-bond geometry (Å, °)

*D*—H⋯*A*	*D*—H	H⋯*A*	*D*⋯*A*	*D*—H⋯*A*
C3—H3⋯F3^i^	0.95	2.45	3.263 (2)	144
C7—H7*A*⋯N2^ii^	0.99	2.59	3.567 (2)	170
C7—H7*B*⋯F6^iii^	0.99	2.28	3.264 (2)	172
C10—H10⋯F2^iv^	0.95	2.53	3.373 (2)	148
C11—H11*B*⋯F4^iv^	0.99	2.44	3.355 (2)	154
C13—H13⋯F4^iv^	0.95	2.34	3.193 (2)	149

Symmetry codes: (i) 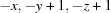; (ii) 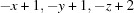; (iii) 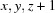; (iv) 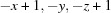.

**Fig. 1.**
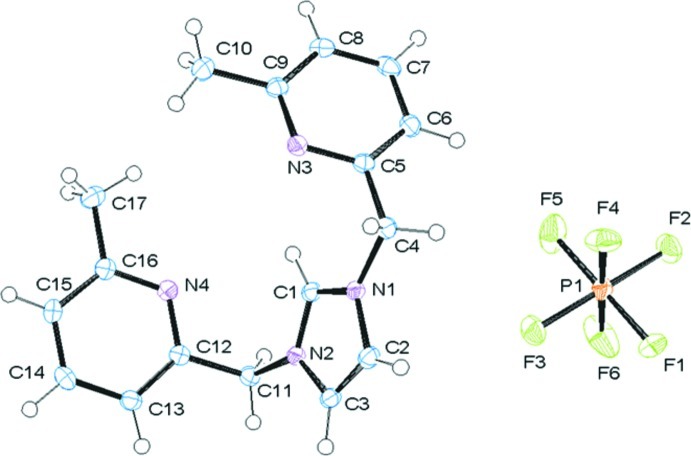
The molecular structure of the title compound, showing displacement ellipsoids drawn at the 50% probability level.

**Fig. 2.**
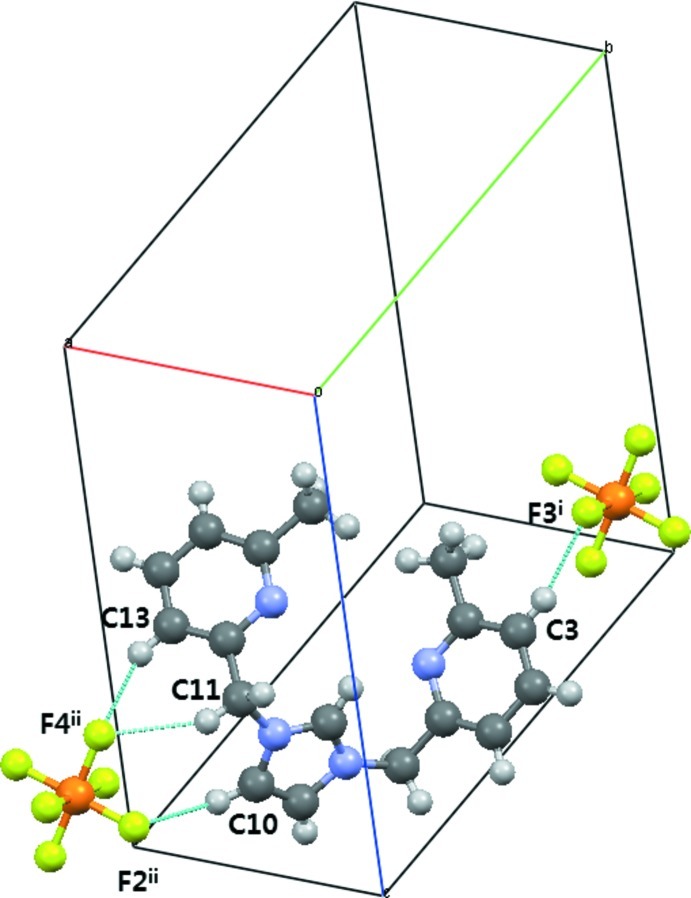
C—H···F interactions (dotted lines) in the title compound. [Symmetry code: (i) -x, -y + 1, -z + 1; (ii) -x + 1, -y, -z + 1]

## supplementary crystallographic information

### Comment

*N*,*N*'-Disubstituted imidazolium salts are of importance because stable and isolable *N*-heterocyclic carbenes (NHCs) can be easily prepared by deprotonation of the imidazolium salts with a strong base. *N*-Heterocyclic carbenes (NHCs) have been a topic of extensive research due to their practical applications in many catalytic processes, *e.g.*, Pd–catalysed Heck–, Suzuki–coupling and Ru-based Grubbs catalyses (Frenzel *et al.*, 1999; Enders *et al.*, 1996; Scholl *et al.*, 1999). We previously synthesized an *N*,*N*'-disubstituted imidazolium salt, 1,3-bis[(6-methyl-2-pyridinyl)methyl]imidazolium bromide, and reported its crystal structure (Kim *et al.*, 2009)). The title compound was obtained by the anion exchange of the C_17_H_19_N_4_^+^ Br^-^ with NH_4_PF_6_. Here we report the crystal structure of the title compound,1,3-bis[(6-methyl-2-pyridinyl)methyl]imidazolium hexafluorophosphate (Fig. 1).

The asymmetric unit of the title compound consists the C_17_H_9_N_4_ cation and PF_6_ anion. Each of two 6–methylpyridine rings is rotated out of the imidazole plane, with dihedral angle of N1/C2–C6 of 55.83 (9)° and N4/C12–C16 of 11.32 (9)°. The molecular packing is stabilized by four different intermolecular C—H···F hydrogen bonds in the structure. (Table 1 & Fig. 2).

### Experimental

Synthesis of 1,3-Bis[(6-methylpyridin-2-yl)-1*H*-imidazolium hexafluorophosphate: A mixture of 1,3-Bis[(6-methylpyridin-2-yl)-1*H*-imidazolium bromide (Kim *et al.*, 2009). (2.16 g, 6.01 *x* 10^–3^ mol) and NH_4_PF_6_ (0.980 g, 6.01 *x* 10^–3^ mol) were dissolved in acetonitrile (35 ml) and the reaction mixture was stirred at room temperature for 19 h. After the solution was filtered the solvent were removed by high-vacuum rotary evaporation. The filtrate was dried under the reduced pressure to afford a brown solid in 95% yield. Single crystals were obtained by Et_2_O diffusion into a CHCl_3_ solution of the compound.

Spectroscopic analysis: 1H NMR (CDCl_3_, 400 MHz): δ8.95 (s, H, CH), 7.62 (t, 2H, J =7.8 Hz, CH), 7.47 (d, 2H, J = 2 Hz, CH), 7.31 (s, 2H, CH), 7.28(d, 2H J = 10 Hz, CH), 7.14 (d, 2H, J = 7.8 Hz, CH), 5.35 (s, 4H, CH_2_), 2.50 (s, 6H, CH_3_). 13 C NMR (CDCl_3_, 100 MHz): δ159.0 (s, C), 150.9 (s, C), 137.9 (s, CH), 136.1 (s, CH), 123.7 (s, CH), 122.4 (s, CH), 120.5 (s, CH), 54.5 (s, CH2), 24.4 (s, CH_3_).

### Refinement

Hydrogen atoms were treated as riding on their parent carbon atoms, with *U*_iso_(H) = 1.2 to .5 *U*_eq_(C).

### Crystal data

**Table:**

C_17_H_19_N_4_^+^·PF_6_^−^	*Z* = 2
*M**_r_* = 424.33	*F*(000) = 436
Triclinic, *P*1	*D*_x_ = 1.557 Mg m^−^^3^
Hall symbol: -P 1	Mo *K*α radiation, λ = 0.71073 Å
*a* = 6.3839 (3) Å	Cell parameters from 3700 reflections
*b* = 12.0353 (5) Å	θ = 1.7–26.5°
*c* = 12.8006 (5) Å	µ = 0.22 mm^−^^1^
α = 108.039 (2)°	*T* = 100 K
β = 96.091 (2)°	Block, colorless
γ = 100.593 (2)°	0.16 × 0.07 × 0.07 mm
*V* = 905.12 (7) Å^3^	

### Data collection

**Table:**

Bruker APEXII diffractometer	2898 reflections with *I* > 2σ(*I*)
Radiation source: fine-focus sealed tube	*R*_int_ = 0.032
Graphite monochromator	θ_max_ = 26.5°, θ_min_ = 1.7°
θ/2πhi scans	*h* = −8→7
Absorption correction: multi-scan (*SADABS*; Sheldrick, 1996)	*k* = −15→15
*T*_min_ = 0.965, *T*_max_ = 0.986	*l* = −16→16
19206 measured reflections	4 standard reflections every 10 min
3700 independent reflections	intensity decay: 0.0%

### Refinement

**Table:**

Refinement on *F*^2^	Primary atom site location: structure-invariant direct methods
Least-squares matrix: full	Secondary atom site location: difference Fourier map
*R*[*F*^2^ > 2σ(*F*^2^)] = 0.039	Hydrogen site location: inferred from neighbouring sites
*wR*(*F*^2^) = 0.133	H-atom parameters constrained
*S* = 0.97	*w* = 1/[σ^2^(*F*_o_^2^) + (0.1*P*)^2^] where *P* = (*F*_o_^2^ + 2*F*_c_^2^)/3
3700 reflections	(Δ/σ)_max_ < 0.001
269 parameters	Δρ_max_ = 0.35 e Å^−^^3^
0 restraints	Δρ_min_ = −0.45 e Å^−^^3^

### Special details

**Table:**

Geometry. All e.s.d.'s (except the e.s.d. in the dihedral angle between two l.s. planes) are estimated using the full covariance matrix. The cell e.s.d.'s are taken into account individually in the estimation of e.s.d.'s in distances, angles and torsion angles; correlations between e.s.d.'s in cell parameters are only used when they are defined by crystal symmetry. An approximate (isotropic) treatment of cell e.s.d.'s is used for estimating e.s.d.'s involving l.s. planes.
Refinement. Refinement of *F*^2^ against ALL reflections. The weighted *R*-factor *wR* and goodness of fit *S* are based on *F*^2^, conventional *R*-factors *R* are based on *F*, with *F* set to zero for negative *F*^2^. The threshold expression of *F*^2^ > σ(*F*^2^) is used only for calculating *R*-factors(gt) *etc*. and is not relevant to the choice of reflections for refinement. *R*-factors based on *F*^2^ are statistically about twice as large as those based on *F*, and *R*- factors based on ALL data will be even larger.

### Fractional atomic coordinates and isotropic or equivalent isotropic displacement parameters (Å^2^)

**Table:**

	*x*	*y*	*z*	*U*_iso_*/*U*_eq_	
P1	0.02678 (7)	0.17116 (4)	0.22243 (4)	0.02100 (17)	
F1	−0.20955 (18)	0.16206 (10)	0.25487 (10)	0.0319 (3)	
F2	0.26436 (17)	0.18066 (10)	0.18965 (10)	0.0307 (3)	
F3	0.09571 (18)	0.30854 (9)	0.30069 (11)	0.0335 (3)	
F4	0.1109 (2)	0.13509 (11)	0.32678 (10)	0.0383 (3)	
F5	−0.0381 (2)	0.03350 (11)	0.14681 (12)	0.0517 (4)	
F6	−0.05468 (19)	0.21073 (14)	0.12030 (11)	0.0472 (4)	
N1	0.4837 (2)	0.09220 (12)	0.78766 (12)	0.0161 (3)	
N2	0.2781 (2)	0.42854 (12)	0.85867 (12)	0.0172 (3)	
N3	0.5463 (2)	0.18196 (13)	0.59095 (12)	0.0178 (3)	
N4	0.3855 (2)	0.21935 (12)	0.92229 (12)	0.0159 (3)	
C1	0.3009 (3)	0.54548 (17)	0.73429 (16)	0.0246 (4)	
H1A	0.3075	0.6315	0.7669	0.037*	
H1B	0.2319	0.5167	0.6557	0.037*	
H1C	0.4478	0.5319	0.7397	0.037*	
C2	0.1717 (3)	0.47883 (15)	0.79610 (14)	0.0181 (4)	
C3	−0.0512 (3)	0.46979 (15)	0.78953 (15)	0.0201 (4)	
H3	−0.1230	0.5075	0.7462	0.025 (5)*	
C4	−0.1665 (3)	0.40628 (16)	0.84576 (16)	0.0218 (4)	
H4	−0.3181	0.3993	0.8416	0.027 (5)*	
C5	−0.0567 (3)	0.35243 (15)	0.90892 (15)	0.0205 (4)	
H5	−0.1320	0.3067	0.9477	0.023 (5)*	
C6	0.1643 (3)	0.36706 (15)	0.91398 (14)	0.0173 (4)	
C7	0.2937 (3)	0.31832 (16)	0.98693 (14)	0.0199 (4)	
H7A	0.4132	0.3841	1.0371	0.032 (6)*	
H7B	0.1998	0.2888	1.0340	0.023 (5)*	
C8	0.3746 (3)	0.17802 (15)	0.81261 (14)	0.0162 (4)	
H8	0.3008	0.2054	0.7604	0.017 (5)*	
C9	0.5053 (3)	0.15701 (16)	0.96936 (15)	0.0194 (4)	
H9	0.5382	0.1678	1.0465	0.033 (6)*	
C10	0.5668 (3)	0.07800 (15)	0.88527 (15)	0.0189 (4)	
H10	0.6517	0.0227	0.8920	0.030 (5)*	
C11	0.5118 (3)	0.02583 (15)	0.67421 (14)	0.0187 (4)	
H11A	0.3680	−0.0104	0.6259	0.028 (5)*	
H11B	0.5827	−0.0401	0.6767	0.043 (7)*	
C12	0.6464 (3)	0.10626 (15)	0.62463 (14)	0.0164 (4)	
C13	0.8589 (3)	0.10113 (16)	0.61417 (15)	0.0198 (4)	
H13	0.9250	0.0475	0.6396	0.021 (5)*	
C14	0.9729 (3)	0.17597 (16)	0.56576 (15)	0.0210 (4)	
H14	1.1185	0.1743	0.5573	0.020 (5)*	
C15	0.8716 (3)	0.25257 (16)	0.53018 (15)	0.0199 (4)	
H15	0.9460	0.3038	0.4960	0.022 (5)*	
C16	0.6582 (3)	0.25440 (15)	0.54479 (14)	0.0185 (4)	
C17	0.5434 (3)	0.33675 (18)	0.50650 (18)	0.0277 (4)	
H17A	0.5198	0.3113	0.4248	0.042*	
H17B	0.6318	0.4190	0.5378	0.042*	
H17C	0.4035	0.3337	0.5319	0.042*	

### Atomic displacement parameters (Å^2^)

**Table:**

	*U*^11^	*U*^22^	*U*^33^	*U*^12^	*U*^13^	*U*^23^
P1	0.0187 (3)	0.0230 (3)	0.0248 (3)	0.0041 (2)	0.0065 (2)	0.0125 (2)
F1	0.0232 (6)	0.0308 (6)	0.0450 (7)	0.0050 (5)	0.0158 (5)	0.0147 (5)
F2	0.0196 (6)	0.0401 (7)	0.0371 (7)	0.0072 (5)	0.0100 (5)	0.0176 (5)
F3	0.0274 (6)	0.0206 (6)	0.0519 (8)	0.0073 (5)	0.0026 (5)	0.0117 (5)
F4	0.0493 (8)	0.0459 (8)	0.0417 (8)	0.0283 (6)	0.0193 (6)	0.0317 (6)
F5	0.0393 (8)	0.0334 (8)	0.0601 (10)	−0.0049 (6)	0.0220 (7)	−0.0123 (6)
F6	0.0257 (7)	0.0908 (11)	0.0386 (8)	0.0116 (7)	0.0034 (6)	0.0427 (8)
N1	0.0154 (7)	0.0146 (7)	0.0194 (8)	0.0032 (6)	0.0042 (6)	0.0073 (6)
N2	0.0157 (8)	0.0177 (7)	0.0187 (8)	0.0043 (6)	0.0038 (6)	0.0062 (6)
N3	0.0169 (8)	0.0183 (8)	0.0181 (8)	0.0037 (6)	0.0024 (6)	0.0062 (6)
N4	0.0135 (7)	0.0172 (7)	0.0188 (7)	0.0036 (6)	0.0030 (6)	0.0086 (6)
C1	0.0240 (10)	0.0272 (10)	0.0275 (10)	0.0082 (8)	0.0058 (8)	0.0142 (8)
C2	0.0203 (9)	0.0157 (8)	0.0171 (9)	0.0050 (7)	0.0031 (7)	0.0035 (7)
C3	0.0178 (9)	0.0170 (9)	0.0239 (9)	0.0054 (7)	0.0004 (7)	0.0047 (7)
C4	0.0139 (9)	0.0185 (9)	0.0292 (10)	0.0027 (7)	0.0031 (7)	0.0036 (8)
C5	0.0192 (9)	0.0164 (9)	0.0249 (10)	0.0027 (7)	0.0070 (7)	0.0052 (7)
C6	0.0185 (9)	0.0151 (8)	0.0182 (9)	0.0049 (7)	0.0044 (7)	0.0041 (7)
C7	0.0225 (10)	0.0211 (9)	0.0177 (9)	0.0076 (7)	0.0061 (7)	0.0066 (7)
C8	0.0133 (8)	0.0170 (8)	0.0190 (9)	0.0024 (7)	0.0016 (7)	0.0081 (7)
C9	0.0172 (9)	0.0235 (9)	0.0207 (9)	0.0045 (7)	0.0021 (7)	0.0124 (7)
C10	0.0158 (9)	0.0203 (9)	0.0246 (10)	0.0046 (7)	0.0032 (7)	0.0130 (8)
C11	0.0193 (9)	0.0177 (9)	0.0197 (9)	0.0062 (7)	0.0064 (7)	0.0048 (7)
C12	0.0169 (9)	0.0158 (8)	0.0141 (8)	0.0041 (7)	0.0007 (7)	0.0025 (7)
C13	0.0172 (9)	0.0211 (9)	0.0213 (9)	0.0054 (7)	0.0021 (7)	0.0075 (7)
C14	0.0147 (9)	0.0239 (9)	0.0229 (10)	0.0047 (7)	0.0037 (7)	0.0055 (8)
C15	0.0190 (9)	0.0220 (9)	0.0178 (9)	0.0013 (7)	0.0052 (7)	0.0067 (7)
C16	0.0188 (9)	0.0204 (9)	0.0151 (9)	0.0040 (7)	0.0024 (7)	0.0051 (7)
C17	0.0272 (11)	0.0284 (10)	0.0345 (11)	0.0087 (8)	0.0067 (9)	0.0186 (9)

### Geometric parameters (Å, º)

**Table:**

P1—F5	1.5883 (13)	C4—H4	0.9500
P1—F6	1.5933 (12)	C5—C6	1.381 (2)
P1—F3	1.5951 (12)	C5—H5	0.9500
P1—F4	1.5980 (12)	C6—C7	1.501 (2)
P1—F1	1.6003 (12)	C7—H7A	0.9900
P1—F2	1.6095 (12)	C7—H7B	0.9900
N1—C8	1.327 (2)	C8—H8	0.9500
N1—C10	1.377 (2)	C9—C10	1.345 (3)
N1—C11	1.473 (2)	C9—H9	0.9500
N2—C2	1.343 (2)	C10—H10	0.9500
N2—C6	1.346 (2)	C11—C12	1.506 (3)
N3—C16	1.341 (2)	C11—H11A	0.9900
N3—C12	1.350 (2)	C11—H11B	0.9900
N4—C8	1.326 (2)	C12—C13	1.388 (2)
N4—C9	1.380 (2)	C13—C14	1.388 (3)
N4—C7	1.482 (2)	C13—H13	0.9500
C1—C2	1.496 (3)	C14—C15	1.376 (2)
C1—H1A	0.9800	C14—H14	0.9500
C1—H1B	0.9800	C15—C16	1.398 (2)
C1—H1C	0.9800	C15—H15	0.9500
C2—C3	1.399 (2)	C16—C17	1.501 (2)
C3—C4	1.375 (3)	C17—H17A	0.9800
C3—H3	0.9500	C17—H17B	0.9800
C4—C5	1.391 (2)	C17—H17C	0.9800
			
F5—P1—F6	91.57 (8)	C5—C6—C7	120.87 (16)
F5—P1—F3	178.51 (7)	N4—C7—C6	112.82 (14)
F6—P1—F3	89.86 (7)	N4—C7—H7A	109.0
F5—P1—F4	89.92 (8)	C6—C7—H7A	109.0
F6—P1—F4	178.50 (8)	N4—C7—H7B	109.0
F3—P1—F4	88.65 (7)	C6—C7—H7B	109.0
F5—P1—F1	90.45 (7)	H7A—C7—H7B	107.8
F6—P1—F1	89.57 (6)	N4—C8—N1	108.81 (14)
F3—P1—F1	89.98 (6)	N4—C8—H8	125.6
F4—P1—F1	90.59 (7)	N1—C8—H8	125.6
F5—P1—F2	89.65 (7)	C10—C9—N4	106.99 (15)
F6—P1—F2	90.28 (6)	C10—C9—H9	126.5
F3—P1—F2	89.92 (6)	N4—C9—H9	126.5
F4—P1—F2	89.57 (6)	C9—C10—N1	107.26 (15)
F1—P1—F2	179.82 (6)	C9—C10—H10	126.4
C8—N1—C10	108.43 (15)	N1—C10—H10	126.4
C8—N1—C11	124.97 (14)	N1—C11—C12	111.69 (14)
C10—N1—C11	126.59 (14)	N1—C11—H11A	109.3
C2—N2—C6	118.40 (15)	C12—C11—H11A	109.3
C16—N3—C12	118.01 (15)	N1—C11—H11B	109.3
C8—N4—C9	108.50 (14)	C12—C11—H11B	109.3
C8—N4—C7	127.20 (14)	H11A—C11—H11B	107.9
C9—N4—C7	124.26 (15)	N3—C12—C13	122.89 (16)
C2—C1—H1A	109.5	N3—C12—C11	115.65 (15)
C2—C1—H1B	109.5	C13—C12—C11	121.46 (15)
H1A—C1—H1B	109.5	C14—C13—C12	118.66 (16)
C2—C1—H1C	109.5	C14—C13—H13	120.7
H1A—C1—H1C	109.5	C12—C13—H13	120.7
H1B—C1—H1C	109.5	C15—C14—C13	118.86 (17)
N2—C2—C3	121.23 (17)	C15—C14—H14	120.6
N2—C2—C1	117.52 (16)	C13—C14—H14	120.6
C3—C2—C1	121.25 (16)	C14—C15—C16	119.50 (17)
C4—C3—C2	120.01 (17)	C14—C15—H15	120.2
C4—C3—H3	120.0	C16—C15—H15	120.2
C2—C3—H3	120.0	N3—C16—C15	122.06 (16)
C3—C4—C5	118.67 (17)	N3—C16—C17	117.27 (16)
C3—C4—H4	120.7	C15—C16—C17	120.66 (16)
C5—C4—H4	120.7	C16—C17—H17A	109.5
C6—C5—C4	118.44 (17)	C16—C17—H17B	109.5
C6—C5—H5	120.8	H17A—C17—H17B	109.5
C4—C5—H5	120.8	C16—C17—H17C	109.5
N2—C6—C5	123.21 (16)	H17A—C17—H17C	109.5
N2—C6—C7	115.87 (15)	H17B—C17—H17C	109.5

### Hydrogen-bond geometry (Å, º)

**Table:**

*D*—H···*A*	*D*—H	H···*A*	*D*···*A*	*D*—H···*A*
C3—H3···F3^i^	0.95	2.45	3.263 (2)	144
C7—H7*A*···N2^ii^	0.99	2.59	3.567 (2)	170
C7—H7*B*···F6^iii^	0.99	2.28	3.264 (2)	172
C10—H10···F2^iv^	0.95	2.53	3.373 (2)	148
C11—H11*B*···F4^iv^	0.99	2.44	3.355 (2)	154
C13—H13···F4^iv^	0.95	2.34	3.193 (2)	149

Symmetry codes: (i) −*x*, −*y*+1, −*z*+1; (ii) −*x*+1, −*y*+1, −*z*+2; (iii) *x*, *y*, *z*+1; (iv) −*x*+1, −*y*, −*z*+1.
